# Zinc is an inhibitor of the LdtR transcriptional activator

**DOI:** 10.1371/journal.pone.0195746

**Published:** 2018-04-10

**Authors:** Fernando A. Pagliai, Lei Pan, Danilo Silva, Claudio F. Gonzalez, Graciela L. Lorca

**Affiliations:** Department of Microbiology and Cell Science, Genetics Institute, Institute of Food and Agricultural Sciences, University of Florida, Gainesville, FL, United States of America; Florida International University, UNITED STATES

## Abstract

LdtR is a master regulator of gene expression in *Liberibacter asiaticus*, one of the causative agents of citrus greening disease. LdtR belongs to the MarR-family of transcriptional regulators and it has been linked to the regulation of more than 180 genes in *Liberibacter* species, most of them gathered in the following Clusters of Orthologous Groups: cell motility, cell wall envelope, energy production, and transcription. Our previous transcriptomic evidence suggested that LdtR is directly involved in the modulation of the zinc uptake system genes (*znu*) in the closely related *L*. *crescens*. In this report, we show that LdtR is involved in the regulation of one of the two encoded zinc uptake mechanisms in *L*. *asiaticus*, named *znu*_2_. We also show that LdtR binds zinc with higher affinity than benzbromarone, a synthetic effector inhibitory molecule, resulting in the disruption of the LdtR:promoter interactions. Using site-directed mutagenesis, electrophoretic mobility shift assays (EMSAs), and isothermal titration calorimetry, we identified that residues C28 and T43 in LdtR, located in close proximity to the Benz1 pocket, are involved in the interaction with zinc. These results provided new evidence of a high-affinity effector molecule targeting a key player in *L*. *asiaticus*’ physiology and complemented our previous findings about the mechanisms of signal transduction in members of the MarR-family.

## Introduction

Huanglongbing (HLB) or citrus greening disease is predominantly caused by *Liberibacter asiaticus*, a Gram-negative, phloem-limited, fastidious α-proteobacterium. This bacterium is transmitted from plant to plant by the phloem-sap feeder psyllid named *Diaphorina citri* Kuwayama [1] and has generated a devastation in the citrus industry over the last 10 years [1–3]. Among the symptoms usually observed in the plant are yellow blotchy mottle, asymmetrical leaf chlorosis, premature fruit drop, as well as reduced fruit and juice quality [4–6]. Some of these distinguishing symptoms are also associated with the limitation of nutrients [1,7]. Unfortunately, the lack of traditional genetic and molecular tools, plus the inability to maintain cultures of this bacterium under laboratory settings, has hampered the ability to understand in depth the physiology of *L*. *asiaticus*. Most of the knowledge about this citrus pathogen has been inferred from the annotation derived of its genomic sequence [8]. However, few biochemical studies have addressed the role of key targets, such as the regulatory proteins LdtR and PrbP [9–12], the chaperone-interacting protein LotP [13], the salicylate hydroxylase SahA [14], or the periplasmic zinc binding protein ZnuA_2_ [15].

Since *L*. *asiaticus* only resides in the citrus phloem sap, it is expected that this space contains all the nutrients required by this bacterium to grow. Recently, in a characterization of the composition of the phloem sap of *Citrus sinensis* (var. pineapple sweet orange), it was found that the phloem sap is rich in flavonoids, hydroxycinnamates, nucleotides, macro- and micro-nutrients [16]. The most abundant macro-nutrients were calcium and magnesium, whereas the most abundant micro-nutrients were zinc and manganese [16]. It is not surprising that the deficiency in any of these nutrients is generally associated with low crop yields [17]. In this regard, the deficiency of zinc in citrus produces leaf chlorosis, a condition that resembles the symptoms of citrus greening disease [18]. Despite these symptomatic similarities, the role of macro- and micro-nutrients in the physiology of *L*. *asiaticus*, or any close-related species, has been scarcely studied [19].

Zinc is one of the most abundant transition metals in living organisms [20]. Within biological systems it prevails as a divalent cation (Zn^2+^) and has been predicted to play catalytic, structural, and regulatory roles in up to 6% of prokaryotic proteins [21,22]. Bacteria often require specific mechanisms for the uptake of zinc since its charge and hydrophilic characteristics are incompatible with passive diffusion through the cellular membrane [23]. One specific zinc import mechanism, denominated Znu (Zinc uptake) system, was discovered and well-characterized in *Escherichia coli* [24]. The Znu system displays the characteristics of the ATP-binding cassette transporters (ABC) where ZnuA acts a periplasmic metallochaperone that recognizes and binds zinc, ZnuB is the integral membrane permease, and ZnuC is the ATPase subunit that provides energy for the uptake process at expenses of ATP.

One of the sixteen ABC transporter systems up-regulated when *L*. *asiaticus* is located in the plant compared to the psyllid [25] is homologous to the ZnuABC system. In addition, in our recent transcriptomic study conducted in *L*. *crescens* it was observed that most of the components of the Znu systems significantly changed their expression when the LdtR transcriptional regulator was chemically inactivated [10]. LdtR is the only member of the MarR-family of transcriptional regulators encoded in the genome of *Liberibacter* species. In both *L*. *asiaticus* and *L*. *crescens*, LdtR has been associated with the control of the expression of genes classified in the following Clusters of Orthologous Groups (COGs): cell motility, cell wall biogenesis, transcription, and energy production [10]. The goal of this study was to analyze the molecular interactions between zinc and LdtR to establish the foundations for the use of zinc alone or in combination with other inhibitors, to target and inactivate LdtR. Using a combination of DNA binding assays, site-directed mutagenesis, as well as thermal melting and calorimetry analyses, we were able to elucidate the amino acids in LdtR involved in the interactions with Zn^2+^.

## Materials and methods

### Bacterial strains and growth conditions

The plasmids and strains used in this study are listed in Table 1. The expression vectors for protein purification were propagated into *Escherichia coli* DH5α and BL21(DE3). *E*. *coli* cultures were grown in LB broth (Fisher Scientific, Pittsburgh, PA, USA) supplemented with ampicillin (100 μg/ml) under aerobic conditions (250 rpm) at 37°C. *L*. *crescens* BT-1 was cultured at 26°C with aeration (200 rpm) in modified BM7 media [10]. All the chemicals and antibiotics were purchased from Sigma-Aldrich (St. Louis, MO, USA).

**Table 1:** List of strains and plasmids used in this study.

Name	Relevant genotype	Origin/reference
**Bacterial Strains**		
*E*. *coli* DH5α	φ80 d*lacZ*ΔM15Δ(*lacZYA-argF*)U169 *recA1 endA1 hsdR17* (rk^-^. mk^+^) *supE44 thi-1 gyrA relA1*.	Laboratory stock
*E*. *coli* BL21 (DE3)	*F–ompT gal dcm lon hsdSB*(*rB- mB-*) *λ*(*DE3* [*lacI lacUV5-T7 gene 1 ind1 sam7 nin5]*).	Life Technologies
*L*. *crescens* BT-1	Standard strain (Wild Type).	[26]
**Plasmid**		
p15TV-L	Expression vector for purification of proteins by nickel affinity cromatography. Ap^R^.	(Addgene plasmid # 26093)

### DNA manipulation and gene cloning

Standard methods were utilized for DNA isolation, enzyme digestion, and agarose gel electrophoresis [27]. PCR products were purified using QIAquick purification kit (Qiagen, Valencia, CA, USA), whereas the plasmids were isolated using QIAprep Spin Miniprep kit (Qiagen). Site-directed mutagenesis was conducted using the QuikChange II Site-Directed Mutagenesis Kit (Agilent Technologies, Santa Clara, CA, USA) according to the manufacturers recommended protocol. Successful mutations were confirmed via DNA sequencing using T7 primers. The primers used in this study are listed in Table 2.

**Table 2:** Oligonucleotides used in this study.

Primer	Oligonucleotide sequence (5’ → 3’)
**Site-directed mutagenesis**	
LdtR_C28S_Fw	tctggtctatatgtggaaagtttgcgtttggttgagcga
LdtR_C28S_Rv	tcgctcaaccaaacgcaaactttccacatatagaccaga
LdtR_E33A_Fw	ggaatgcttgcgtttggttgcgcgattacacagaagtcttttgg
LdtR_E33A_Rv	ccaaaagacttctgtgtaatcgcgcaaccaaacgcaagcattcc
**EMSAs**	
PznuA_1__Fw	ggtgttagacaagctgatc
PznuA_1__Rv^a^	cctgctctagctacactagac
PznuA_2__Fw	gtttacaaataatctcaaattacac
PznuA_2__Rv^a^	atgccgacatgggaatata
PldtP_Fw^a^	ccagagaaagacccataggc
PldtP_Rv	tacagcgtttaaatcgtttttg
**Sequencing**	
T7	taatacgactcactataggg
T7 term	gctagttattgctcagcgg

^a^ biotin labeled

### Protein purification

Protein expression and purification was conducted as previously described with modifications [28]. Briefly, the hexa his-tagged WT LdtR, as well as the mutants, were overexpressed in *E*. *coli* BL21-Star(DE3) cells (Life Technologies, Grand Island, NY, USA). The cells were grown in LB medium with aeration (250 rpm) at 37°C until OD600 reached 0.5. The overexpression of the proteins was induced by adding 0.5 mM IPTG and the cells incubated in the shaker with aeration at 17°C overnight. After harvesting, the cells were washed and suspended in binding buffer (500 mM NaCl, 5% glycerol, 50 mM Tris pH 8.0, 5 mM imidazole, and 0.5 mM TCEP) and the cells lysed in a French press. The cell-free extract was loaded in a metal chelate affinity-column charged with Ni^2+^ (Qiagen). The column was washed with binding buffer supplemented with 25 mM imidazole. After elution (binding buffer supplemented with 250 mM imidazole), the purified proteins in solution were dialyzed for 16 h in 500 mM NaCl, 5% glycerol, 50 mM Tris pH 8.0, 0.5 mM TCEP, and 250 μM EDTA. The proteins were subsequently dialyzed for 24 h in 500 mM NaCl, 5% glycerol, 50 mM Tris pH 8.0, 0.5 mM TCEP. The concentration of the purified proteins was determined with the Bio-Rad protein assay, using bovine serum albumin as a standard reference (Bio-Rad, Hercules, CA).

### Electrophoretic mobility shift assays

DNA shift assays of LdtR, as well as its mutated versions, was carried out as described previously [9]. Fragments of the selected promoters were generated by PCR using biotinylated primers (Table 2). The reaction mix for EMSA contained 1 ng of 5’biotin-labelled DNA probe, 50 nM Tris-HCl pH 7.2, 150 mM KCl, 10 mM MgCl_2_, 0.01% Triton X100, 12.5 ng/μl both Poly(dI-dC) and Poly(dA-dT) nonspecific competitor DNAs, and purified LdtR (0–1000 nM) as indicated. The mix was incubated for 20 min at 37°C and the electrophoresis was conducted at 4°C on 6% acrylamide/bisacrylamide non-denaturing gels in 0.5X Tris-borate EDTA buffer (TBE) pH 8.3. The DNA was transferred to a Hybond-N^+^ membrane (GE Healthcare, Pittsburgh, PA, USA) by electroblotting at 250 mA for 45 min in a Semi Dry Electroblotting System (Fisher Scientific). The detection of the DNA bands was carried out with the Phototope-Star Detection Kit (New England Biolabs, Ipswich, MA, USA). The membranes were exposed to Autoradiography Film (Mid-Sci, St. Louis, MI, USA).

### Isothermal titration calorimetry

The measure of heat exchange for the interaction between LdtR (WT and mutants C28S or T43A) with zinc chloride and/or benzbromarone was conducted on a MicroCal ITC200 system (Malvern Instruments Inc., Westborough, MA, USA). Purified proteins at 50 μM were used. Prior to ITC experiments, each protein was dialyzed for 24 h in 500 mM NaCl, 5% glycerol, 50 mM Tris pH 8.0, 0.5 mM TCEP. The runs were carried out at 28°C and injections of 2 μl of ligand were added to the protein solution stirring at 700 rpm. The mean enthalpies from the injections of the ligand into the buffer were subtracted from the raw titration data before the calculation of the thermodynamic parameters. The titration curves were fitted by a non-linear square method, using Origin 7.0 software (MicroCal) using either one-set or two-set of binding sites. Thermodynamic parameters, such as Δ*H* (reaction enthalpy), *K*_*A*_ (binding constant *K*_*A*_ = 1/*K*_*D*_), and *N* (stoichiometry of the reaction) were calculated. Δ*G* (changes in free energy) and Δ*S* (entropy) were calculated from the *K*_*A*_ and Δ*H* values using the equation Δ*G = –*RT*ln*K*_*A*_ = Δ*H*–TΔ*S*, where T is the absolute temperature and R is the universal molar gas constant.

### Phylogenetic analysis

The protein sequences of LdtR homologs used in this study were retrieved from NCBI database [29] and were aligned using MUSCLE [30].

### Thermal stability assays by differential scanning fluorimetry

Purified LdtR was tested with different concentrations of Zn^2+^ (0–50 μM) as described previously [9,31]. LdtR was diluted in 100 mM Tris buffer pH 8.0 and 150 mM NaCl to a final concentration of 30 μM. The reagent SYPRO orange protein stain (ThermoFisher, Waltham, MA, USA) was added to a final concentration of 5X. Volumes of 25 μl of a mix containing ligand, protein, buffers, and SYPRO reagent were added in a 96-well plate (Bio-Rad) and ran in triplicate. The samples were first incubated at 25°C for 5 min and then heated to 80°C at a rate of 1°C per min. By measuring the increase in the fluorescence of the SYPRO orange reagent, the unfolding of LdtR was monitored in a multicolor real-time PCR detection system (iCycler iQ, Bio-Rad). The generated fluorescence intensities were plotted against temperature for each sample and the denaturation curves were analyzed and fitted using the Boltzmann equation in Microcal Origin 2017 software (OriginLab, Northampton, MA, USA). The midpoint of each denaturation curve of LdtR in presence of Zn^2+^ was calculated and compared to the midpoint value of LdtR in presence of buffer as a control.

### Statistical analyses

The statistical significance of the different melting temperatures of LdtR, as well as the growth rates of *L*. *crescens*, was determined using an analysis of variance (ANOVA) and a Tukey’s HSD *post hoc* test. During the course of all the experiments a *p*-value < 0.05 was considered as statistically significant and α = 0.05 was used for the Tukey’s HSD tests.

## Results

### *Liberibacter* species possess different *znu* gene clusters

**Fig 1:**
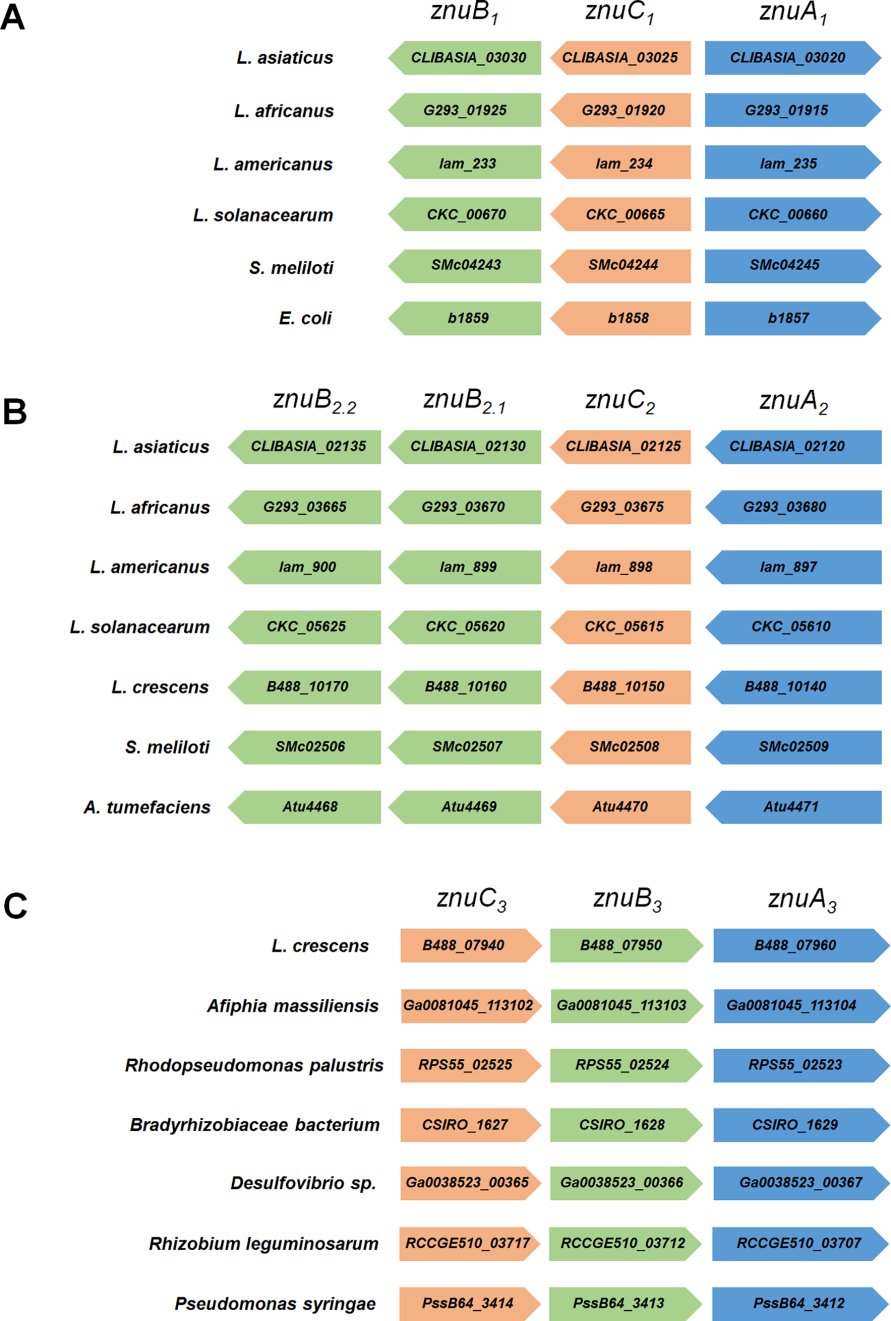
Genomic arrangement of *znu* gene clusters identified in *L*. *asiaticus*. Regulon #1 possesses the genomic arrangement *znuACB* (A), regulon #2 has an arrangement *znuACBB* (B), whereas regulon #3 displays the arrangement *znuCBA* (C). The components of each system are colored as follows: *znuA* (blue), *znuB* (green), and *znuC* (orange). Homologs from *Liberibacter* species, as well as *S*. *meliloti*, *E*. *coli*, *A*. *tumefaciens*, *Afiphia massiliensis*, *Rhodopseudomonas palustris*, *Bradyrhizobiaceae bacterium*, *Desulfovibrio sp*., *Rhizobium leguminosarum*, and *Pseudomonas syringae* were included in the analysis. The arrows represent each homologue (drawn to scale) and describe the direction of their transcription.

The genome of *L*. *asiaticus* psy62 encodes for two gene clusters with homology to the *znuABC* system described in *E*. *coli* and *Sinorhizobium meliloti* [24,32]. One system, named regulon #1 [33], contains three genes with the arrangement *znuACB* (encoded by *CLIBASIA_03020*-*CLIBASIA_03025*-*CLIBASIA_03030* and named *znuA*_*1*_, *znuC*_*1*_, and *znuB*_*1*_, respectively) with *znuA*_*1*_ located downstream and divergently oriented from *znuC*_*1*_*B*_*1*_. This regulon is conserved in the closely related species *L*. *americanus*, *L*. *africanus*, *L*. *solanacearum*, as well as in *S*. *meliloti* and *E*. *coli*; however, it is absent in *L*. *crescens* (Fig 1A). A second cluster, termed regulon #2, contains four genes with the arrangement *znuACBB* (encoded by *CLIBASIA_02120*-*CLIBASIA_02125*-*CLIBASIA_02130*-*CLIBASIA_02135* and named *znuA*_*2*_, *znuC*_*2*_, *znuB*_*2*.*1*_, and *znuB*_*2*.*2*_, respectively). This regulon is conserved in *L*. *americanus*, *L*. *africanus*, *L*. *solanacearum*, *L*. *crescens*, *S*. *meliloti*, and *A*. *tumefaciens*, but absent in *E*. *coli*. Similar to *L*. *asiaticus*, the genome of *L*. *crescens* BT-1 contains two *znu* gene clusters; however, only the regulon #2 is conserved between these two *Liberibacter* species (*znuA*_*2*_*C*_*2*_*B*_*2*.*1*_*B*_*2*.*2*_, encoded by *B488_10140*-*B488_10150*-*B488_10160*-*B488_10170* in *L*. *crescens*, Fig 1B). The regulon #3 identified in *L*. *crescens* displays an alternative *znuC*_*3*_*B*_*3*_*A*_*3*_ arrangement with *znuA*_*3*_ in the 3’-end of the putative operon. This genomic arrangement is conserved in some alpha-proteobacteria, such as *Afiphia massiliensis*, *Rhodopseudomonas palustris*, and *Bradyrhizobiaceae bacterium*, but absent in other *Liberibacter* species. Interestingly, this operon structure is also present in the delta-proteobacteria *Desulfovibrio sp*. and the gamma-proteobacteria *Pseudomonas syringae* (Fig 1C).

### LdtR is involved in the regulation of the *znuA*_*2*_*C*_*2*_*B*_*2*.*1*_*B*_*2*.*2*_ gene cluster

**Fig 2:**
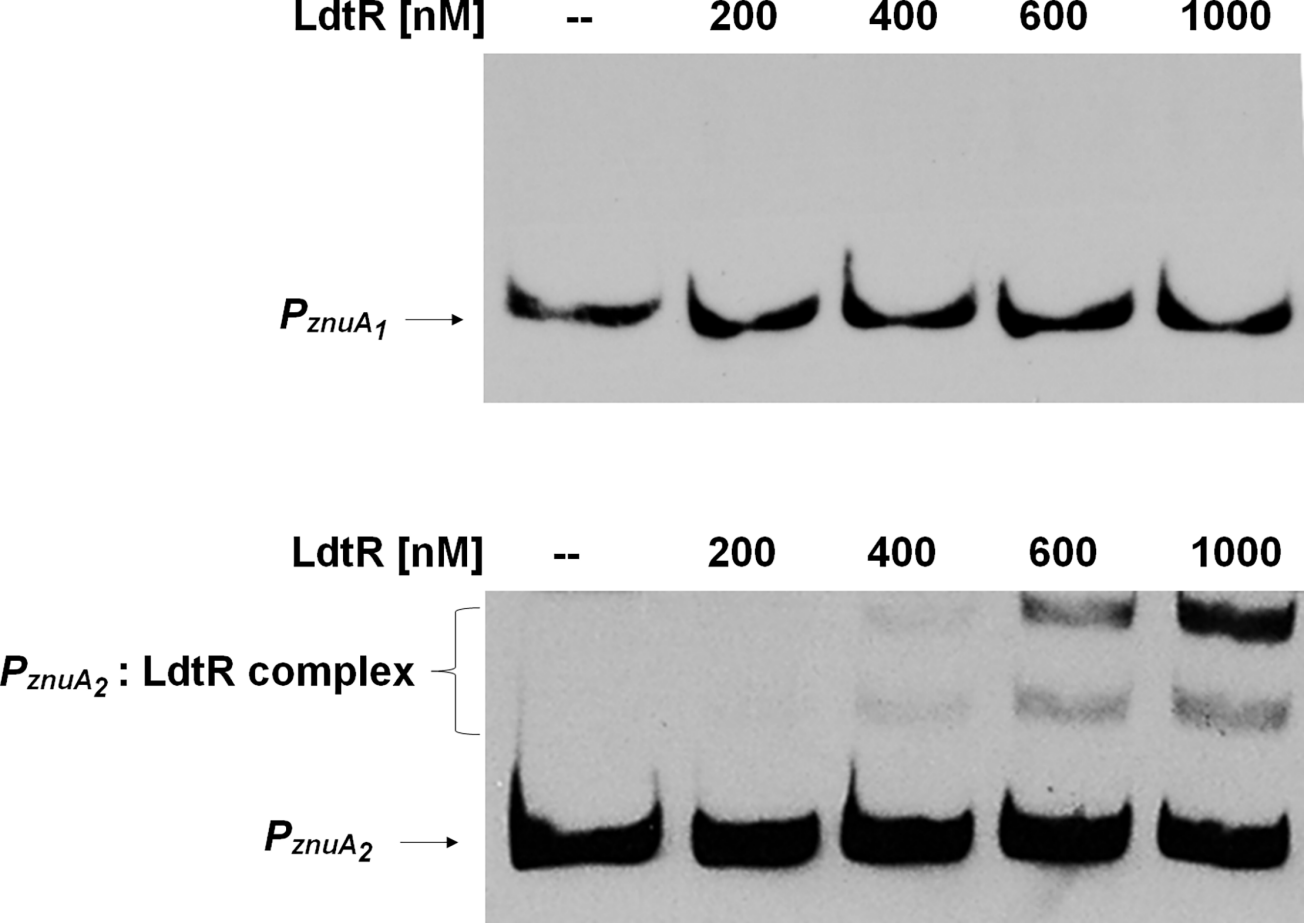
LdtR binds to *P*_*znuA2*_ but not to *P*_*znuA1*_. EMSAs were performed using *znuA* promoters from *L*. *asiaticus*. *P*_*znuA1*_ and *P*_*znuA2*_ probes were incubated with increasing concentration of LdtR (0–1000 nM), as indicated on top of each panel. Protein was not added to the first lane.

It has been demonstrated that in both *E*. *coli* and *S*. *meliloti* the Znu gene cluster is regulated by the zinc uptake regulator Zur [24,33], a member of the ferric uptake regulator (Fur) family. However, no homologs of this regulator have been found in the genome of either *L*. *asiaticus* or *L*. *crescens* [8,26]. These findings suggest that other transcriptional regulator(s) may be involved in metal cations uptake. Our recent transcriptomic studies conducted in *L*. *crescens* BT-1 evidenced that the expression of both *znu* regulons was significantly affected upon the chemical inactivation of LdtR [10]. Although these results suggested that LdtR is involved in the regulation of the *znuABC* systems in *L*. *crescens*, the regulation of the *znuABC* systems has yet to be determined in *L*. *asiaticus*. To elucidate the role of LdtR in the regulation of *znu* gene clusters in *L*. *asiaticus*, biotinylated probes containing the promoter regions of both *znuA*_*1*_ and *znuA*_*2*_ genes were synthesized. Each fragment of DNA spanned approximately 200 bp upstream of either *CLIBASIA_03020* or *CLIBASIA_02120* (named *P*_*znuA1*_ and *P*_*znuA2*_, respectively) and was tested in DNA binding assays as described previously [9]. Although LdtR *in vivo* modulates the expression of *znuA*_2_ *and znuA*_3_ homologues in *L*. *crescens* [10], it was found that LdtR only bound to *P*_*znuA2*_ and not to *P*_*znuA1*_ from *L*. *asiaticus* (Fig 2). Using RegPredict [34], two LdtR binding boxes were identified within *P*_*znuA2*_; the first located between the bases -28(TATTTTATTAAA-*n5*-TATAATTGAATA)+1, and the second located between the bases -191(ATAAAGAACTGA-*n2*-TAAGTTTTTTTT)-167, with respect to the translational start point. Each inverted repeat shows a separation of 2–5 nucleotides between each segment which may explain the lower affinity for LdtR, similar distribution was observed in the *ldtR* promoter (*P*_*ldtR*_*)* [9]. Using the same approach, no LdtR binding boxes were predicted within the promoter of *znuA*_*1*_. These predictions are consistent with the DNA binding assays of LdtR over *znu* promoters, and suggest that in *L*. *asiaticus*, LdtR may be involved in the regulation of the *znuA*_*2*_*C*_*2*_*B*_*2*.*1*_*B*_*2*.*2*_ gene cluster, whereas another transcriptional regulator, yet to be identified, may be involved in the regulation of the *znuA*_*1*_*C*_*1*_*B*_*1*_ operon.

### Metal cations disrupt the binding of LdtR to *P*_*znuA2*_

**Fig 3:**
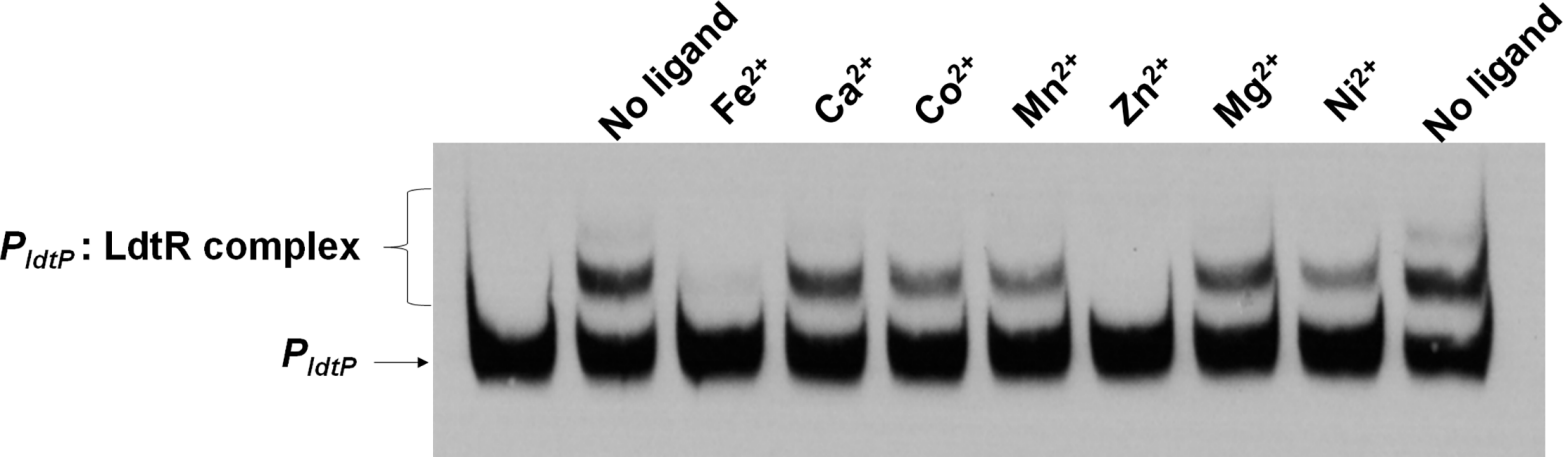
Effect of metal cofactors in the interaction between LdtR and *P*_*ldtP*_. EMSAs were carried out using 400 nM LdtR with the addition of 100 μM metal chlorides, as indicated on top of each panel. Protein was not added to the first lane.

**Fig 4:**
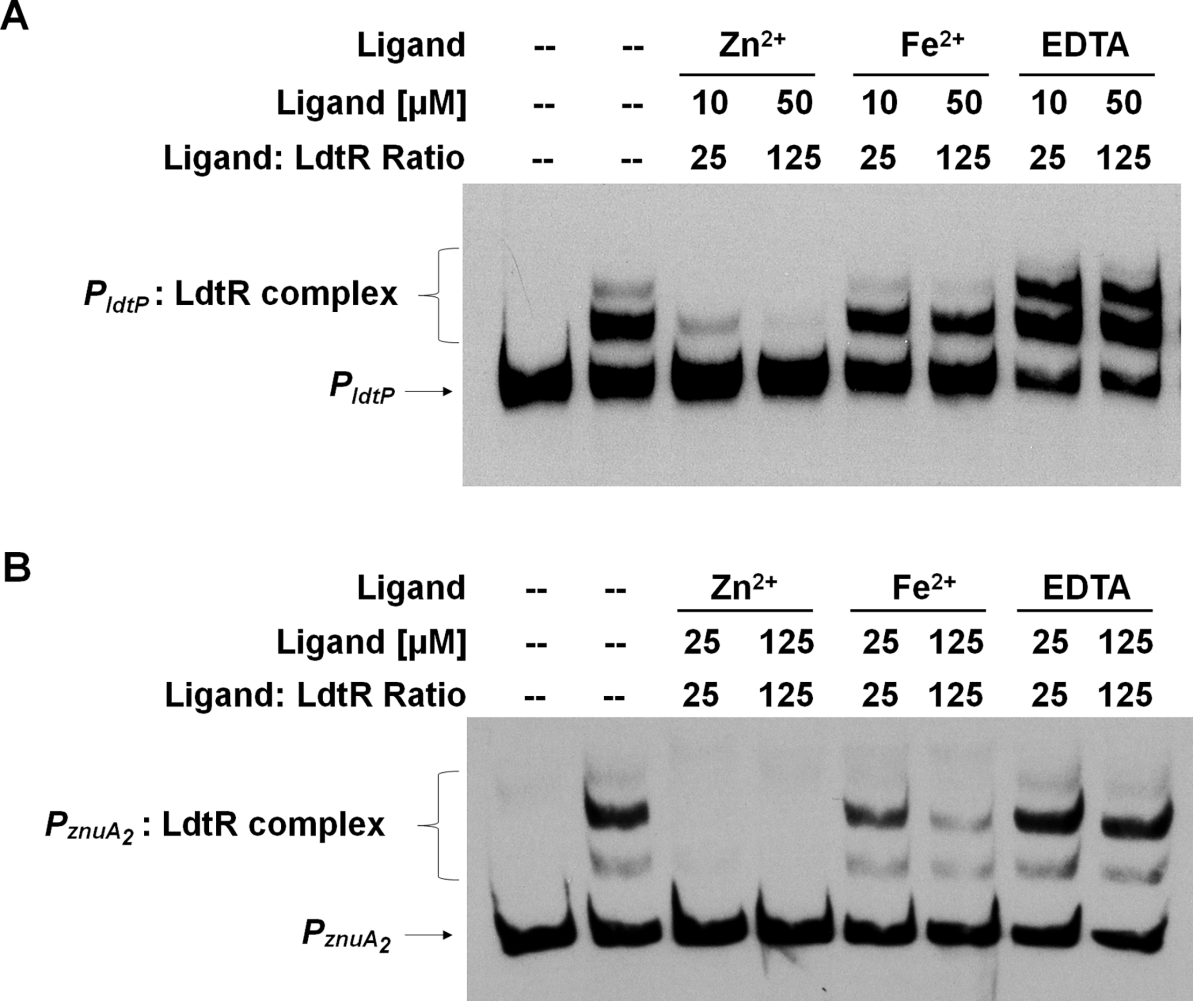
Zinc and EDTA have antagonistic effects on the LdtR interaction with DNA. EMSAs were carried out using 400 nM LdtR on *P*_*ldtP*_ (A) or 1000 nM LdtR on *P*_*znuA2*_ (B) in the absence or presence of different concentrations of Zn^2+^, Fe^2+^, or EDTA. The ligand:LdtR molar ratio is indicated on top of each panel. Protein was not added to the first lane.

Since the *znu* gene cluster has been associated with the uptake of micronutrients such as zinc or manganese [24,35], different metal chlorides were incubated with LdtR to evaluate its DNA binding capabilities on LdtR’s cognate promoter *P*_*ldtP*_ (Fig 3). It was found that Zn^2+^, and in a lesser extent Fe^2+^, were able to disrupt the interaction between LdtR and *P*_*ldtP*_, whereas Ca^2+^, Co^2+^, Mn^2+^, Mg^2+^, and Ni^2+^ did not disrupt the interaction between LdtR and DNA. All the metal cations were added at 100 μM (250-fold ligand:protein molar ratio) in the reaction mix. These results were confirmed by running a dose dependency assay with different concentrations of Zn^2+^ or Fe^2+^. In addition, a combination of the metals with the chelator EDTA was also evaluated. For this purpose, two different DNA fragments were used; a high-affinity binding site (*P*_*ldtP*_) and a low-affinity binding site (*P*_*znuA2*_), as described above. It was observed that Zn^2+^ partially disrupted the interaction between LdtR and *P*_*ldtP*_ at lower concentrations (10 μM, 25-fold ligand:protein molar ratio) with a full disruption at 50 μM (125-fold), confirming the Zn^2+^: LdtR interactions (Fig 4A). At low Fe^2+^ concentrations (10 μM), no disruption of the *P*_*ldtP*_:LdtR interaction was observed, whereas at 50 μM only the larger DNA-protein complex was disrupted. Interestingly, the addition of EDTA improved the binding of LdtR to *P*_*ldtP*_, suggesting the protein may still contains some associated metal molecules after the purification process. Comparable results were obtained when using the *znuA*_*2*_ promoter (Fig 4B). 25 μM Zn^2+^ (25-fold ligand:protein molar ratio) disrupted the interaction between LdtR and *P*_*znuA2*_, whereas Fe^2+^ partially disrupted the larger DNA-protein complex at high concentrations (125 μM). The addition of EDTA did not improve the binding of LdtR to *P*_*znuA2*_. Similar to that observed in Fig 2, LdtR did not bind to *P*_*znuA1*_, despite the addition of Zn^2+^, EDTA, or a combination of both (data not shown). Altogether, these results suggest that zinc modulates the activity of LdtR, acting as an inhibitor of DNA binding, similar to that observed with phloretin or benzbromarone [9].

### LdtR has two binding sites for Zn^2+^

In our previous publication, a model of LdtR was used to identify the benzbromarone binding pocket named Benz1 [28], equivalent to the salicylate pocket SAL1 in MTH313 from *Methanobacterium thermoautotrophicum* [36]. During the course of those studies, it was determined that the residues T43, L61, and F64 were involved in the binding of benzbromarone. However, none of these amino acids are commonly found among zinc binding sites in proteins [37,38]. Due to the high structural identity between transcriptional regulators of the MarR-family, a manual search in the PDB database was conducted to identify MarR-family members with Zn^2+^ in their crystal structures. Three models of LdtR were then generated, using AdcR from *Streptococcus pneumoniae* (PDB# 3TGN, [39]), PA3341 from *Pseudomonas aeruginosa* (PDB# 2FBH), and STK_17100 from *Sulfolobus tokodaii* (PDB# 2YR2). For every generated model, the residues surrounding the zinc ions were located within Benz1, suggesting that this ligand binding pocket may interact with Zn^2+^ (data not shown).

**Fig 5:**
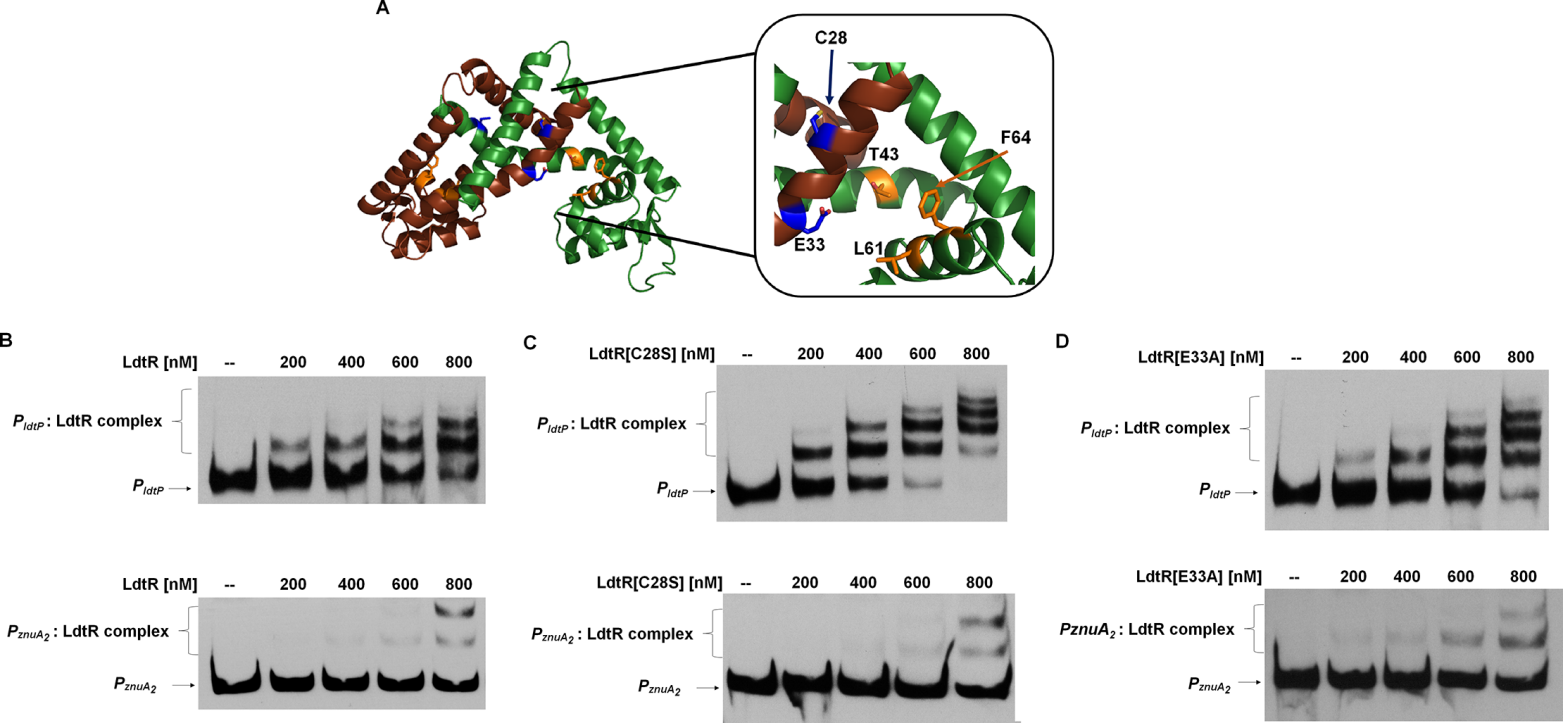
Identification of amino acids in LdtR involved in Zn^2+^ interaction. (A) Close view of the Benz1 pocket identified in LdtR [28]. The model is shown in cartoon representation with monomers A and B colored in brown and green, respectively. The identified amino acids are depicted as sticks and colored in orange (Benz1) or blue for residues C28 and E33. (B) Titration of LdtR binding with *P*_*ldtP*_ and *P*_*znuA2*_ probes by EMSAs. (C) Titration of LdtR(C28S) binding with *P*_*ldtP*_ and *P*_*znuA2*_ probes by EMSAs. (D) Titration of LdtR(E33A) binding with *P*_*ldtP*_ and *P*_*znuA2*_ probes by EMSAs. The concentration of LdtR used in the binding assay is shown on top of each panel. Protein was not added to the first lane.

*In silico* docking of zinc into the LdtR model was performed using Swiss-dock and Docking server [40,41]. However, no molecules of Zn^2+^ were successfully docked into the LdtR dimeric protein. Therefore a manual search along the model of LdtR [28] was conducted to identify potential residues capable of interacting with Zn^2+^, such as cysteine, histidine, aspartic acid and glutamic acid. A similar manual search was positively used to identify two iron binding sites in TstR, another MarR-family member from *Lactobacillus brevis* [42]. It was found that LdtR possesses a cysteine (C28) and a glutamic acid (E33) residue in the α1 helix, in close proximity (less than 10 Å) to the Benz1 (Fig 5A). Although E33 is highly conserved among rhizobiales, C28 is only conserved in some *Liberibacter* species (S1 Fig). To determine if these newly identified amino acids are involved in the interaction with Zn^2+^, site-directed mutagenesis was conducted in LdtR. The mutated proteins were purified in the same conditions as described above for the wild type (WT). Since several studies have shown that mutations in residues involved in ligand recognition may affect the DNA binding capabilities of MarR-family members, the mutated proteins were first tested in EMSAs. LdtR mutants in C28 and E33 displayed similar affinity for *P*_*ldtP*_ and *P*_*znuA2*_ compared to the WT LdtR [9,10]. Under the current DNA gel shift conditions, approximately 50% binding of LdtR(C28S) to *P*_*ldtP*_ was reached at 400 μM of the protein (Fig 5C), whereas for LdtR(E33A) it was reached at 600 nM (Fig 5D). Approximately 50% of binding of either mutant to *P*_*znuA2*_ was achieved using 800 μM of protein (Fig 5C and 5D). These results are similar to those obtained with the WT LdtR (Fig 5B), and suggest that mutations in these residues do not affect the ability of LdtR to bind DNA.

### LdtR mutants C28S, T43A, and L61A have decreased binding of Zn^2+^

**Fig 6:**
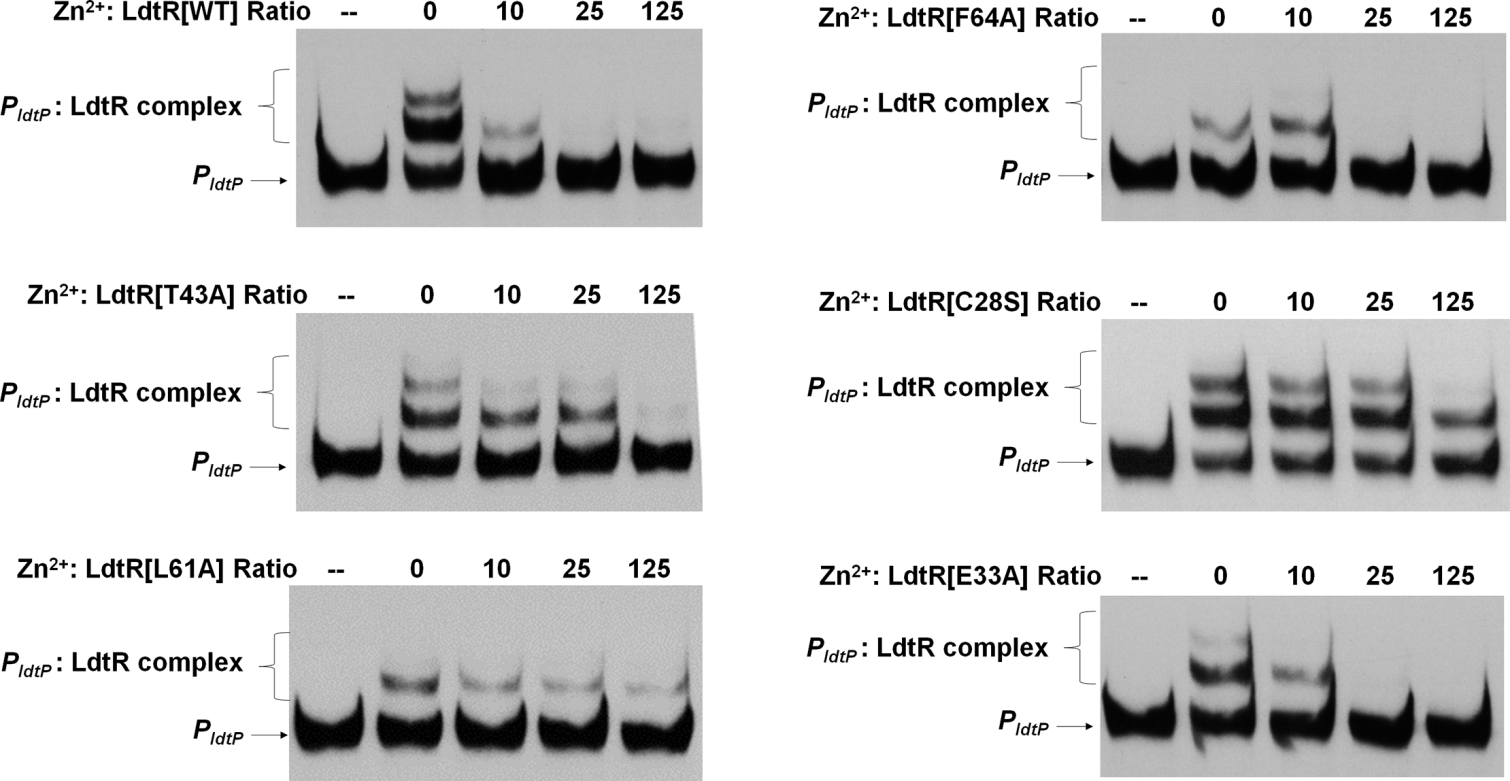
Mutations in residues C28 and T43 and L61 affect the interaction of LdtR with Zn^2+^. The *P*_*ldtP*_ probe was incubated with LdtR WT or the LdtR mutants T43A, L61A, F64A, C28S, and E33A in the absence or presence of increasing concentrations of zinc. Since some LdtR mutants have different affinities for *P*_*ldtP*_ [28] and the Zn^2+^:LdtR molar ratio was kept constant, as indicated on top of each panel. Protein was not added to the first lane.

To determine the role of crucial residues in LdtR for sensing ligands, WT LdtR, mutants in Benz1 pocket [28], as well as the newly described C28S and E33A mutants, were evaluated in DNA binding assays at the conditions where approximately 50% of binding to *P*_*ldtP*_ was reached. The binding of WT LdtR to DNA, as well as mutant E33A, was partially disrupted at 10-fold Zn^2+^:protein molar ratio, whereas a full disruption of the DNA binding was completed at a 25- fold Zn^2+^:protein molar ratio (Fig 6). The mutant L61A required 10-fold molar excess to disrupt most of the DNA:protein interaction, but a full disruption was not observed even when the zinc was added in a 125-fold molar excess, similar to what has been observed for benzbromarone [28]. Although for the mutant F64A, the bigger DNA:protein complex was not observed, a 25-fold molar excess of zinc was able to disrupt the DNA:protein interaction. The mutant T43A required a 125-fold molar excess of Zn^2+^ to fully disrupt the DNA:protein interaction (Fig 6). Notably, mutant C28S showed little to no disruption of DNA when Zn^2+^ was added in a 125-fold molar excess (Fig 6). These results suggest that mutations in residues C28, T43, and L61 affect the interaction of LdtR with Zn^2+^ *in vitro*.

### Mutations of C28 and T43 residues in LdtR decreased affinity for Zn^2+^

Isothermal Titration Calorimetry was used for the determination of the thermodynamic parameters of LdtR:Zn^2+^ interactions for WT LdtR as well as for the C28S, T43A, and L61A mutants. A WT LdtR, in which the 6x histidine tag has been removed, was included as control. For all tested proteins, an exothermal heat exchange was observed in the titration of LdtR with Zn^2+^. No differences were observed in the titrations with zinc in the presence or absence of the 6x histidine tag in LdtR. The titration data for WT LdtR was best fitted using a model of “two sets of sites”, whereas for LdtR mutants C28S and T43A, the data was best fitted using the “one set of sites” model (S2 Fig). Unfortunately, the titration data of LdtR mutant L61A with zinc did not fit under any model and the thermodynamic parameters could not be determined. The thermodynamic parameters of the LdtR:Zn^2+^ interactions are summarized in Table 3. The stoichiometry of the interaction for site #1 was 1.5 moles of Zn^2+^ per mole of monomer LdtR, whereas for site #2, it was 0.4 moles of Zn^2+^ per mole of monomer LdtR. The stoichiometry of Zn^2+^ in site #2 is similar to what was observed for LdtR and benzbromarone [28], where the ligand binds to one pocket in the LdtR dimer. The dissociation constants (*K*_*D*_) for LdtR were 7.6 ± 2.8 μM for site #1 and 2.8 ± 0.2 μM for site #2, both in the low micromolar range.

**Table 3:** Thermodynamic parameters of for the calorimetric titration of LdtR with Zn^2+^.

LdtR		*N*	*K*_*D*_ [μM]	Δ*H* [cal/mol]	Δ*S* [cal/mol/deg]	Δ*G* [kcal/mol]
WT ^a^	Site 1	1.5 ± 0.3	7.6 ± 2.8	-9.3E+04 ± 8.8E+04	-10.1 ± 0.9	-7.6 ± 2.7
WT ^a^	Site 2	0.4 ± 0.1	2.8 ± 0.2	2.0E+04 ± 7.1E+03	49.7 ± 27.7	-7.4 ± 1.3
WT ^b^	Site 1	2.1 ± 0.2	3.3 ± 1.5	-2.2E+03 ± 1.3E+02	18 ± 1.1	-7.6 ± 0.5
WT ^b^	Site 2	0.3 ± 0.1	0.4 ± 0.3	-5.8E+02 ± 1.5E+02	32.1 ± 1.6	-10.2 ± 0.4
C28S	–	0.6 ± 0.4	32.8 ± 6.6	-2.3E+04 ± 1.9E+04	-44.2 ± 25.2	-6.4 ± 0.5
T43A	–	0.5 ± 0.2	58.9 ± 26.1	-6.4E+04 ± 3.9E+04	-147.9 ± 77.2	-5.9 ± 0.6

^a^ WT LdtR containing 6x histidine tag

^b^ WT LdtR without 6X histidine tag

For the LdtR mutants C28S and T43A, each dissociation constant was also in the micromolar range, but significantly higher when compared to LdtR (32.8 ± 6.6 and 58.9 ± 26.1 μM, respectively). The stoichiometry of the reaction was 0.6 moles of Zn^2+^ per mole of monomer C28S mutant and 0.5 moles of Zn^2+^ per mole of monomer T43A mutant. Altogether, these results suggest the presence of two high-affinity sites in WT LdtR for Zn^2+^, with the residues C28 and T43 mediating its interaction in one of the binding sites.

### Zn^2+^ destabilizes LdtR by binding into the Benz1 pocket

A fluorescent based assay [28,43] was used to determine the thermal stability of LdtR when bound to increasing concentrations of Zn^2+^ (ranging from 0.1 to 1.6 metal:protein molar ratio). It was observed that LdtR displayed a denaturation midpoint (*Tm*) of 44.1 ± 0.04°C, whereas the addition of the metal decreased the thermal stability of LdtR in a concentration dependent manner (S4 Fig). At low zinc:LdtR molar ratio (0.3:1) the melting temperature significantly decreased by almost 4°C, whereas at higher ligand:protein molar ratios (0.5:1) a dramatic decrease of the LdtR thermal stability of more than 10°C was observed. These results correlated with the thermal destabilization induced by benzbromarone [28]; however, Zn^2+^ has a much stronger destabilization effect over LdtR and does so at lower ligand:protein molar ratios.

### Zn^2+^ and benzbromarone have additive effects on LdtR:DNA interactions

**Fig 7:**
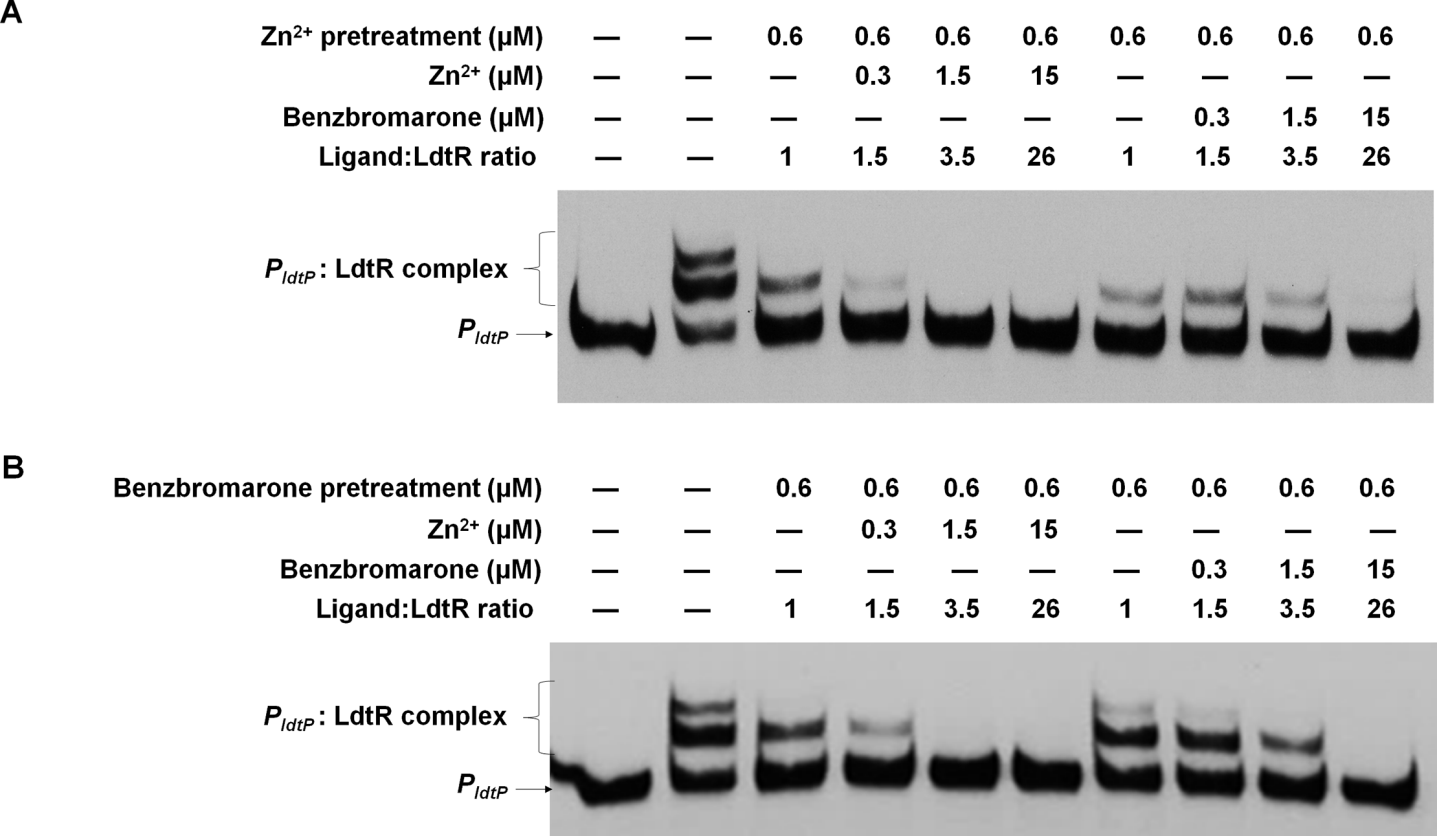
Zn^2+^ and benzbromarone disrupt the binding of LdtR to DNA. The combined effect of both ligands was tested in EMSA. LdtR was preincubated with 0.6 μM Zn^2+^ (A) or benzbromarone (B), in a 1:1 ligand:protein molar ratio. Subsequently, the protein was either incubated with increasing concentrations of either Zn^2+^ or benzbromarone (0.3–15 μM), as indicated on top of each panel. Protein was not added to the first lane.

Both Zn^2+^ and benzbromarone were found to bind LdtR with high affinity, resulting in the disruption of its interaction with the DNA promoters. To determine if there is any synergistic or additive effect of these chemicals, EMSAs combining these two compounds were performed. To do this, LdtR was first incubated with Zn^2+^ in a 1:1 molar ratio and then was titrated with increasing concentrations of either Zn^2+^ or benzbromarone (Fig 7A). It was observed that the addition of Zn^2+^ in a 1:1 molar ratio disrupted the higher LdtR:DNA complex. A full disruption of LdtR:*P*_*ldtP*_ was observed at 3.5-fold ligand:protein molar ratio while benzbromarone required a molar excess of 26-fold to fully disrupt LdtR:*P*_*ldtP*_ interaction. A similar assay was conducted with the exception that LdtR was pretreated with equimolar amounts of benzbromarone (Fig 7B). The addition of benzbromarone at a 1:1 molar ratio also disrupted the higher protein-DNA complex, while a full disruption was observed when benzbromarone was added in a 26-fold molar excess. The subsequent addition of 3.5-fold molar excess of Zn^2+^ fully disrupted the LdtR-DNA interaction. These results suggest that Zn^2+^ and benzbromarone bind into the same binding site in LdtR, but Zn^2+^ does so with higher affinity than benzbromarone.

It was also tested if benzbromarone is able to outcompete zinc in isothermal titration calorimetry experiments. First, LdtR was fully saturated with Zn^2+^ and a subsequent titration with benzbromarone was conducted. It was observed that when the metal cofactor is bound to LdtR, the enthalpic contribution caused by the binding of benzbromarone was minimal and the data could not be fitted under any model, suggesting that Zn^2+^ binds into the Benz1 pocket with higher affinity than benzbromarone (S3 Fig).

### Zn^2+^ enhances growth inhibition by benzbromarone in the *L*. *crescens* model

The chemical inactivation of LdtR in the presence of phloretin, benzbromarone, and zinc observed *in vitro* was validated *in vivo* using *L*. *crescens*. This closely-related bacterium was used as a surrogate model due to the impossibility in maintaining axenic cultures of *L*. *asiaticus* under laboratory settings. The effect of LdtR inhibitors on *L*. *crescens* growth parameters was evaluated by determining the growth rate constant (*k*) and the mean generation time (*g*). First, different concentrations of zinc chloride were added to the BM7 modified media (50–1000 μM) to determine the toxicity of the metal. It was found that 50 and 100 μM Zn^2+^ did not affect the duplication time of the bacteria (approximately 27 h, Table 4). However, the addition of 200 μM Zn^2+^ significantly increased (*p* < 0.05) the duplication time of *L*. *crescens* by 4 h, whereas the addition of 500 μM increased the duplication time by 6 h (*p* < 0.05). The addition of 1000 μM was toxic and no growth was observed (Table 4). Next, the combined effect of different LdtR inhibitors [9] on *L*. *crescens* growth was determined at sublethal concentrations of the chemicals (200 μM Zn^2+^, 25 μM phloretin, or 70 μM benzbromarone). The duplication time of *L*. *crescens* increased by 8 h when 25 μM phloretin was added to the media. The addition of 200 μM Zn^2+^ and 25 μM phloretin resulted in an additive effect, with an increase of 11.6 h in the duplication time of *L*. *crescens*. Similarly, the addition of 70 μM benzbromarone increased the duplication time by 9 h, while the combined addition of Zn^2+^ and benzbromarone increased the duplication time of *L*. *crescens* by 22 h. Altogether, these results confirmed that Zn^2+^, phloretin, and benzbromarone act as inhibitors of LdtR.

**Table 4:** The effect of Zn^2+^, phloretin, and benzbromarone in the growth parameters of *L*. *crescens*.

Ligand [μM]			Growth rate constant, *k*	Mean generation	*p*<0.05
Zn^2+^	phloretin	benzbromarone	(generation/h)^a^	time (h)^b^	
—	—	—	0.0366 ± 0.0006	27.3	
50	—	—	0.0369 ± 0.0011	27.1	
100	—	—	0.0363 ± 0.0009	27.6	
200	—	—	0.0322 ± 0.0008	31	*
500	—	—	0.0298 ± 0.002	33.6	*
1000	—	—	0.0036 ± 0.0005	282.8	*
—	25	—	0.0284 ± 0.006	35.3	*
200	25	—	0.0258 ± 0.0009	38.9	*
—	—	70	0.0275 ± 0.0012	36.5	*
200	—	70	0.0207 ± 0.0031	49	*

^a^ Growth rate constant (*k*) was calculated from the plot of *log*_*2*_OD_600_ versus time

^b^ The mean generation time was calculated as 1/*k*

## Discussion

Bacteria have acquired specific and non-specific transport systems for zinc uptake [23]. Among the specific mechanisms is the high-affinity zinc import complex encoded by the *znuABC* gene cluster [24]. This system shares the characteristics of the ATP-binding cassette transporters; a periplasmic metallochaperone (ZnuA), the membrane permease (ZnuB), and the ATPase subunit (ZnuC). Among *Liberibacter* species, at least three different gene clusters have been identified with the characteristics of the ZnuABC system. At the level of the protein sequences, the components of the Znu_2_ and Znu_3_ clusters are phylogenetically closer. *L*. *asiaticus* encodes for two *znu* gene clusters, named *znu*_1_ and *znu*_2_ [8]. This gene arrangement is conserved in all *Liberibacter* species with the exception of *L*. *crescens*, which lacks *znu*_1_ but encodes an alternative *znu*_3_ gene cluster [26]. Other plant pathogens, such as *Agrobacterium vitis*, *A*. *tumefaciens*, *Erwinia amylovora*, *Pseudomonas syringae* pv. tomato, and *Xanthomonas axonopodi*s pv. citri also encode for two *znu* gene clusters. Their biological role has not been established yet, except in *A*. *tumefaciens* [44], where the *znuABC* genes are negatively regulated by Zur and their expression is induced in response to zinc depletion.

A partial complementation of the ZnuA function in *E*. *coli* was described using homologs of the *znu*_1_ gene cluster from *L*. *asiaticus* and *S*. *meliloti* [33], which suggest an alternative but yet unknown biological role for this transporter system among rhizobiales. In addition, the crystal structure of one of the periplasmic components (ZnuA_2_) from *L*. *asiaticus* was recently solved in a metal-free, as well as Mn^2+^ and Zn^2+^-bound state [15,45]. In these reports, ZnuA_2_ was described to bind both zinc and manganese with low affinities (0.43 and 0.37 mM, respectively) displaying an unusual tetrahedral square pyramidal combination. These findings lead to the hypothesis that ZnuA_2_ could have evolved in plant pathogens to facilitate the transport of both metals, though preferring zinc.

Numerous reports in other microorganisms have described that the expression of the *znu* gene cluster is controlled by the metalloregulatory protein Zur [46–50] which belongs to the Fur-like family of transcriptional regulators [20]. However, neither Zur nor Fur are encoded in the genome of *Liberibacter* species, suggesting the uptake and efflux of these micronutrients may be controlled by an alternative regulatory system. In our previous report, we identified LdtR as modulator of the expression of both *znu* gene clusters in *L*. *crescens* [10]. In this report, we proposed LdtR, a member of the MarR-family, as the transcriptional regulator of the *znu*_2_ gene cluster in *L*. *asiaticus*, whereas the regulatory mechanism for *znu*_1_ gene cluster has yet to be identified.

Other members of the MarR-family have been described to interact with zinc. One of the best studied zinc-related transcriptional regulators is the adhesin competence regulator AdcR from *Streptomyces pneumoniae*. AdcR is a repressor of the high-affinity zinc uptake transporter whose crystal structure revealed that zinc binds with high affinity (*K*_*D1*_ and *K*_*D2*_ < 1 nM) at two distinct sites, one of them equivalent to Benz1, and allosterically activates DNA binding [39,51]. Despite the fact that ZnuA_2_ has been annotated as a part of an iron/manganese transporter there is no biological evidence to confirm its physiological role. Furthermore, LdtR was not affected by iron or manganese in the *in vitro* assays, suggesting this transcriptional regulator may not be involved in sensing either micro-nutrient. Similar to what was observed for AdcR, we established that LdtR has two binding sites for zinc, both displaying high affinity for the metal (*K*_*D*_ 7.6 ± 2.8 and 2.8 ± 0.2 μM). The stoichiometry of the reaction was 1.5:1 for site #1 and 0.5:1 for site #2. These ligand:protein molar ratios are within the range of what was reported in other MarR-family members; 1:1 for zinc: AcdR [51], 1:1 for ethidium bromide: ST1710 or CCCP: ST1710 [52], 2:1 for novobiocin: LVIS553 [53], and 0.5:1 for benzbromarone: LdtR [28].

Despite the number of crystal structures available for MarR-family members, the functional relevance of the ligand binding site has been confirmed only in some cases [28,36,42,53–55]. The affinity values reported here, plus the remarkable decrease in the thermal stability of the protein bound to zinc (almost 12°C decrease in a 1:1 zinc: LdtR molar ratio) provides further evidence of ligand-mediated control of transcription in this family of transcriptional regulators. In the crystal structure of the MarR-family member MTH313 (from *M*. *thermoautotrophicum*), the salicylate ligand binds to the well-studied ligand-binding pocket named SAL1. This binding results in a conformational change of the DNA binding lobes causing a decreased affinity for the DNA [36]. Based on the pocket conservation among this family of regulators, our previous report characterizing Benz1, and the dramatic effect of zinc on LdtR observed *in vitro*, we searched for putative amino acids that can interact with zinc [37,38]. A structure based site-directed mutagenesis in LdtR was used to confirm that the residues C28, T43, and L61 play a pivotal role in the interaction of LdtR with zinc; however, it is possible that the effect is indirect other amino acids may also play a role in the interactions with zinc. Interestingly, T43 and L61 belong to Benz1, whereas C28 is located more than 12 Å away from Benz1, suggesting that C28 belongs to a second and uncharacterized ligand-binding pocket. These observations correlated with our calorimetry experiments where the LdtR: zinc interaction was best fitted in a model of “two sets of sites”, whereas in the individual LdtR mutants, the best fit corresponded to “one set of sites”. The conducted experiments suggest that residues C28, T43, and L61 in LdtR play a role in the interaction with zinc; however, structural studies are required to fully confirm this hypothesis. Interestingly, no biological evidence has confirmed the coordination of zinc ions with an enzyme via a threonine residue. However, in a theoretical study of the interactions between zinc and amino acids, using the density functional theory method, it was suggested that the highest affinity towards zinc occurs via threonine residues [38]. A linear alignment of LdtR homologs found in other rhizobiales showed that residues C28 and T43 are only conserved among *Liberibacter* species, with the exception of *L*. *africanus* that has a phenylalanine instead of a cysteine. In other rhizobiales, T43 is often replaced by isoleucines whereas C28 is occupied by serines (S1 Fig). The conservation of these key amino acids in other *Liberibacter* species provides the opportunity of using high-affinity ligands, such as benzbromarone [9], phloretin [9], or zinc to target the physiological function of LdtR.

In citrus trees infected with *Liberibacter* species it is common to find increased concentrations of potassium and decreased concentrations of calcium, manganese, and zinc [56]. As one of the numerous attempts to control HLB, the enhanced nutritional programme (ENPs) has been executed in order to control this nutrient disparity [57]. Some recent reports indicated that the supplementation of mineral fertilizers containing zinc, iron, or copper, decreased the symptoms in HLB infected trees [58], resulting in an increased productivity in a 5-year span [59]. Recently, a method involving zinc nanoparticles [60] (zinkicide) has been advertised as a potential HLB management option. Furthermore, Hijaz et. al [16] determined that the concentration of zinc in the phloem of ‘Pineapple’ sweet orange was 0.1 mM. Since *L*. *asiaticus* resides in the sieve tube elements of the phloem, our biochemical and *in vivo* assays confirms that the concentration of zinc that exerts an inhibitory effect on LdtR is physiologically relevant. The development of antimicrobial treatments (i.e. zinc) that do not affect the structure of the microbial community and are able to restore the nutrient balance of the plants by targeting specific proteins in *Liberibacter* species, such as LdtR, will provide new and safer options for the HLB eradication.

Understanding the mechanisms employed by *L*. *asiaticus* to adapt and survive in the plant or psyllid host is a feasible approach for the development of new and safer therapeutics to combat citrus greening disease. The evidence presented here, where zinc acts a high-affinity effector molecule for LdtR, indicates that the use of a combination of effector molecules targeting LdtR, such as benzbromarone, phloretin, or zinc, may be an efficient method to impair crucial pathways in *L*. *asiaticus*, providing a safer and novel strategies for the management of HLB infected trees.

## Supporting information

S1 Fig Sequence-based alignment of LdtR and close homologs from the *Rhizobiaceae* family. The alignment was performed using Muscle [30]. The alignment included LdtR homologs from *L*. *americanus* (WP_007556955.1), *L*. *africanus* (WP_047263979.1), *L*. *solanacearum* (WP_034442268.1), *L*. *crescens* (WP_015273508.1), *Sinorhizobium americanum* (WP_064252222.1), *S*. *meliloti* (WP_014526674.1), *Agrobacterium radiobacter* (ACM26108.1), *Rhizobium freirei* (WP_037153623.1), *R*. *etli* (WP_074060777.1), *R*. *leguminosarum* (WP_027685212.1). The secondary structure elements were predicted using PSIPRED [61] and are illustrated on top of the alignment. The α-helices are represented as rectangles and the β-barrels as arrows. The residues from Benz1 pocket [28] as well as the new amino acids identified in this work (C28 and E33) are boxed in a red rectangle.
(PDF)

S2 Fig **Isothermal titration calorimetry data for the binding of zinc into (A) LdtR, (B) LdtR(C28S), (C) LdtR(T43A), and (D) his-tag free LdtR.** Each figure depicts the heat changes (upper panels) and the integrated peak areas (lower panels) from a series of 2-μl injection of the ligand into the protein solution. The experiments were carried out at 28°C.
(PDF)

S3 Fig Isothermal titration calorimetry data for the binding of benzbromarone over zinc-saturated LdtR. Each figure depicts the heat changes (upper panels) and the integrated peak areas (lower panels) from a series of 2-μl injection of the ligand into the protein solution. The experiments were carried out at 28°C.
(PDF)

S4 Fig Binding of zinc to LdtR decreases its thermal stability. The changes in melting temperature (Δ*Tm*) of LdtR were calculated at increasing concentrations of zinc (0–50 μM). Top letters indicate the statistical significance of the changes in melting temperature (a = no significant, b = *p*<0.01).
(PDF)
