# Supplementary material for: Zinc is an inhibitor of the LdtR transcriptional activator
Source: PLoS One. 2018 Apr 10;13(4):e0195746. doi: 10.1371/journal.pone.0195746 (PMC5892913; doi:10.1371/journal.pone.0195746)
Supplement: S1 Fig — The alignment was performed using Muscle [30]. The alignment included LdtR homologs from L. americanus (WP_007556955.1), L. africanus (WP_047263979.1), L. solanacearum (WP_034442268.1), L. crescens (WP_015273508.1), Sinorhizobium americanum (WP_064252222.1), S. meliloti (WP_014526674.1), Agrobacterium radiobacter (ACM26108.1), Rhizobium freirei (WP_037153623.1), R. etli (WP_074060777.1), R. leguminosarum (WP_027685212.1). The secondary structure elements were predicted using PSIPRED [61] and are illustrated on top of the alignment. The α-helices are represented as rectangles and the β-barrels as arrows. The residues from Benz1 pocket [28] as well as the new amino acids identified in this work (C28 and E33) are boxed in a red rectangle. (PDF) [file pone.0195746.s001.pdf]

Supplementary Material

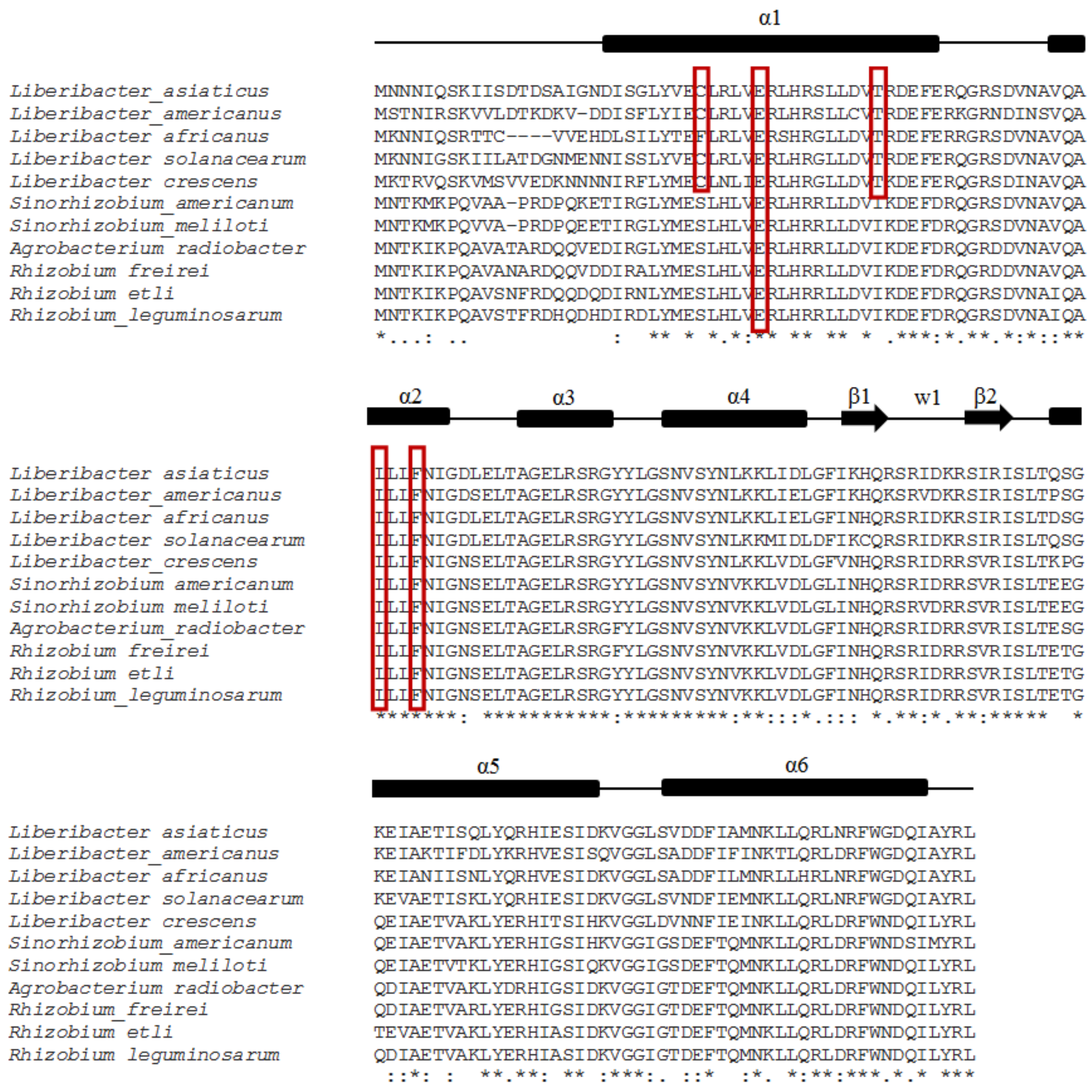

S1 Fig. Sequence-based alignment of LdtR and close homologs from the *Rhizobiaceae* family. The alignment was performed using Muscle (1). The alignment included LdtR homologs from *L. americanus* (WP_007556955.1), *L. africanus* (WP_047263979.1), *L. solanacearum* (WP_034442268.1), *L. crescens* (WP_015273508.1), *Sinorhizobium americanum*

(WP_064252222.1), *S. meliloti* (WP_014526674.1), *Agrobacterium radiobacter* (ACM26108.1), *Rhizobium freirei* (WP_037153623.1), *R. etli* (WP_074060777.1), *R. leguminosarum* (WP_027685212.1). The secondary structure elements were predicted using PSIPRED (2) and are illustrated on top of the alignment. The α-helices are represented as rectangles and the β-barrels as arrows. The residues from Benz1 pocket (3) as well as the new amino acids identified in this work (C28 and E33) are boxed in a red rectangle.
