# Supplementary material for: Zinc is an inhibitor of the LdtR transcriptional activator
Source: PLoS One. 2018 Apr 10;13(4):e0195746. doi: 10.1371/journal.pone.0195746 (PMC5892913; doi:10.1371/journal.pone.0195746)
Supplement: S2 Fig — Isothermal titration calorimetry data for the binding of zinc into (A) LdtR, (B) LdtR(C28S), (C) LdtR(T43A), and (D) his-tag free LdtR. Each figure depicts the heat changes (upper panels) and the integrated peak areas (lower panels) from a series of 2-μl injection of the ligand into the protein solution. The experiments were carried out at 28°C. (PDF) [file pone.0195746.s002.pdf]

**Supplementary Material**

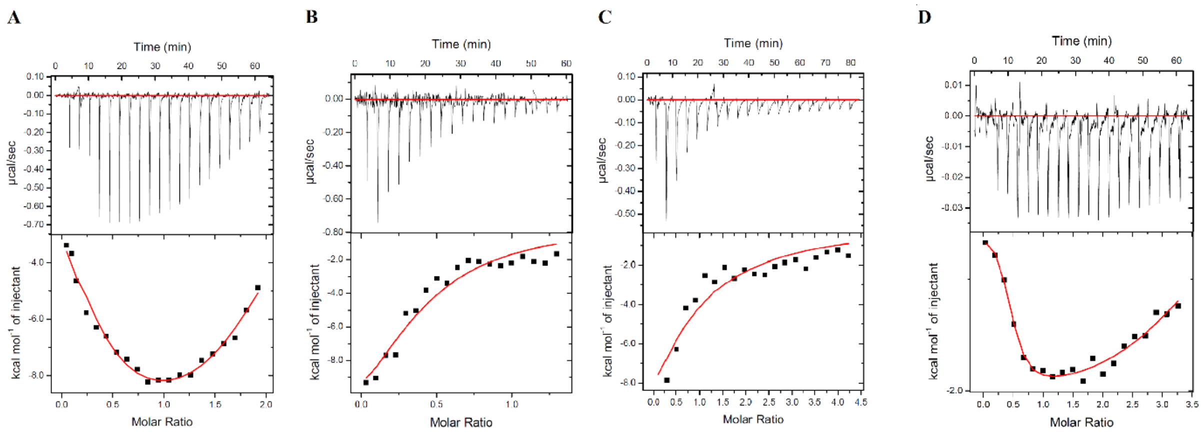


**S2 Fig.** Isothermal titration calorimetry data for the binding of zinc into (A) LdtR, (B) LdtR(C28S), and (C) LdtR(T43A). Each figure depicts the heat changes (upper panels) and the integrated peak areas (lower panels) from a series of 2-µl injection of the ligand into the protein solution. The experiments were carried out at 28°C.
