# Supplementary material for: Zinc is an inhibitor of the LdtR transcriptional activator
Source: PLoS One. 2018 Apr 10;13(4):e0195746. doi: 10.1371/journal.pone.0195746 (PMC5892913; doi:10.1371/journal.pone.0195746)
Supplement: S3 Fig — Each figure depicts the heat changes (upper panels) and the integrated peak areas (lower panels) from a series of 2-μl injection of the ligand into the protein solution. The experiments were carried out at 28°C. (PDF) [file pone.0195746.s003.pdf]

Supplementary Material

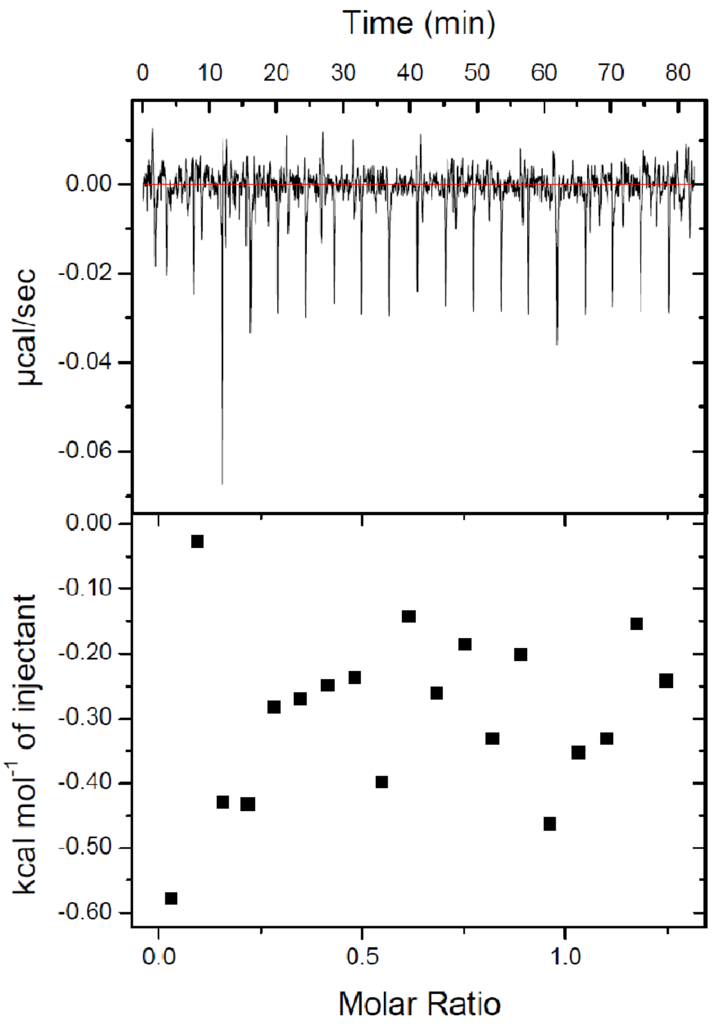

S3 Fig. Isothermal titration calorimetry data for the binding of (A) zinc into his-tag free LdtR or (B) benzbromarone over zinc-saturated LdtR. Each figure depicts the heat changes (upper panels) and the integrated peak areas (lower panels) from a series of 2-µl injection of the ligand into the protein solution. The experiments were carried out at 28°C.
