# Supplementary material for: Zinc is an inhibitor of the LdtR transcriptional activator
Source: PLoS One. 2018 Apr 10;13(4):e0195746. doi: 10.1371/journal.pone.0195746 (PMC5892913; doi:10.1371/journal.pone.0195746)
Supplement: S4 Fig — The changes in melting temperature (ΔTm) of LdtR were calculated at increasing concentrations of zinc (0–50 μM). Top letters indicate the statistical significance of the changes in melting temperature (a = no significant, b = p<0.01). (PDF) [file pone.0195746.s004.pdf]

Supplementary Material

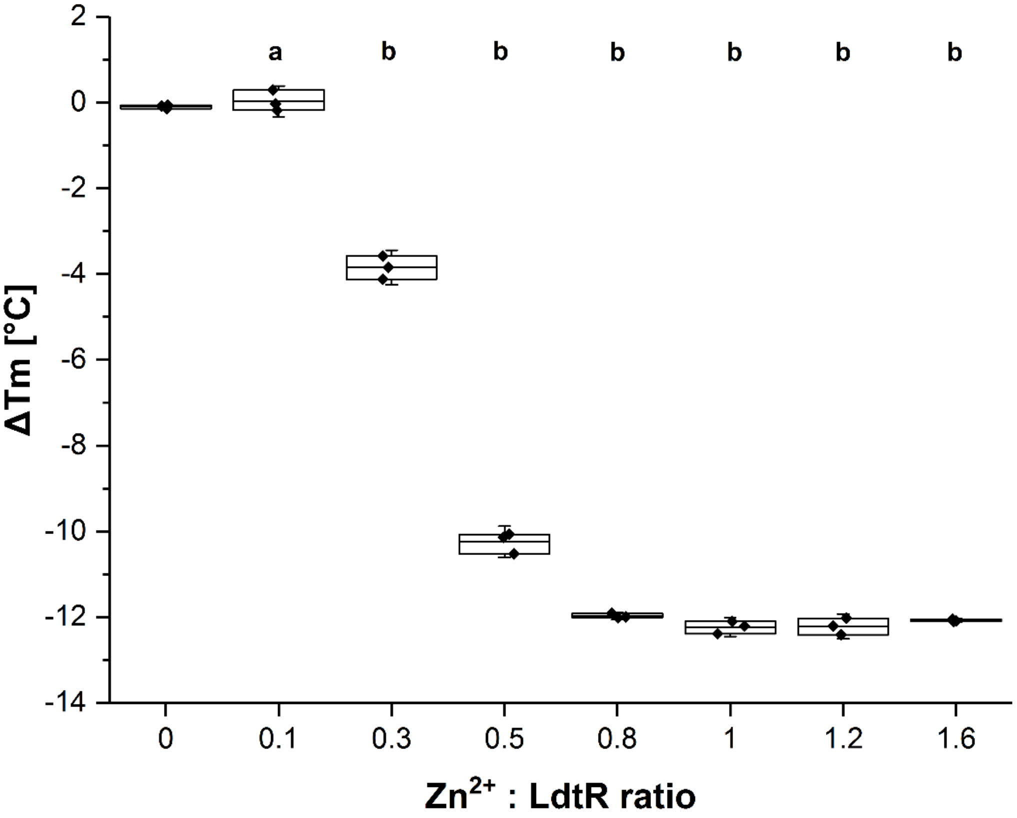

S4 Fig. Binding of zinc to LdtR decreases its thermal stability. The changes in melting temperature (Δ*Tm*) of LdtR were calculated at increasing concentrations of zinc (0-50 µM). Top letters indicate the statistical significance of the changes in melting temperature (a= no significant, b= p<0.01).
